# Investigation on Properties of Raw and Alkali Treated Novel Cellulosic Root Fibres of Zea Mays for Polymeric Composites

**DOI:** 10.3390/polym15071802

**Published:** 2023-04-06

**Authors:** S. Anne Kavitha, R. Krishna Priya, Krishna Prakash Arunachalam, Siva Avudaiappan, Nelson Maureira-Carsalade, Ángel Roco-Videla

**Affiliations:** 1 PG & Research Department of Physics, Holy Cross College (Autonomous), Nagercoil, Manonmaniam Sundaranar University, Tirunelveli 627012, India; 2 Department of Civil Engineering, University College of Engineering, Anna University, Nagercoil 629004, India; 3 Departamento de Ingeniería Civil, Universidad de Concepción, Concepción 4070386, Chile; 4 Centro Nacional de Excelencia para la Industria de la Madera (CENAMAD), Pontificia Universidad Católica de Chile, Av. Vicuña Mackenna 4860, Santiago 8330024, Chile; 5 Department of Physiology, Saveetha Dental College and Hospitals, SIMATS, Chennai 600077, India; 6 Departamento de Ingeniería Civil, Universidad Católica de la Santísima Concepción, Concepción 4090541, Chile; 7 Facultad de Salud y Ciencias Sociales, Universidad de las Américas, Providencia, Santiago 7500975, Chile

**Keywords:** Zea mays (Zm), root fibres, structural, TGA-DTA, light weight, reinforcement

## Abstract

Today, new materials based on natural fibres have been emerging day by day to completely eradicate plastics to favour our environmental nature. In this view, the present work is based on the extraction and characterisation of the novel root fibres of the Zea mays (Zm) plant, grown by the hydroponic method. Both the dried untreated and alkali treated root fibres are investigated using a variety of structural, morphological, thermal, elemental and mechanical tests by subjecting both the samples to p-XRD, FT-IR, SEM-EDAX, TGA-DTA, CHNS and tensile strength analyses. Thermal conductivity of the untreated and treated fibres is found using Lee’s disc experiment. From p-XRD analysis, the Crystallinity Index, Percentage Crystallinity and Crystallite size of the samples are found. FT-IR studies clarify the different vibrational groups associated with the fibre samples. SEM images show that the surface roughness increases for the chemically treated samples, such that it may be effectively utilised as reinforcement for polymeric composites. The diameter of the fibre samples is found using SEM analysis. According to the EDAX spectrum, Zm fibres in both their raw and processed forms have high levels of Carbon (C) and Oxygen (O). The TGA-DTA tests revealed that the samples of natural fibre have good thermal characteristics. CHNS studies show that Carbon content is high for these samples, which is the characteristic of many natural fibres. Chemical analysis is used to ascertain the prepared samples’ chemical makeup. It reveals that both samples have significant amounts of cellulose. The density of the fibres is found to be in the range 0.3–0.6 g/cc, which is much less than any other natural fibre. Therefore, it can be used in light weight applications. From the tensile strength analysis, physical properties such as Young’s modulus and micro-fibril angle are determined. The fibres in the roots exhibit a lower tensile strength. Thus, these fibres can be used in powdered form as reinforcement for natural rubber or epoxy composites. After examining all of its properties, it could be reasonably speculated that Zea mays root fibres can be considered as an efficient reinforcement for various matrices to produce attractive bio-composites.

## 1. Introduction

The prevailing philosophy of the modern world is “Go green and Earth will be clean”. This credo inspires engineers and scientists to develop novel materials based on natural fibres. A group of cells with a small diameter relative to their length may be referred to as a natural fibre. Using a variety of extraction techniques, natural fibres are removed from different plant components. The plant portion from which the fibres are extracted as well as the extraction techniques utilised affect the fibre characteristics. Together with their chemical compositions, other factors that affect the qualities of natural fibres include their physical and chemical morphology, cell wall growth, patterns and thickness, cell size and shape, cross-sectional forms, the distinctiveness of lumens, etc. These fibres are capable of being spun into rope, thread or filaments. To create environmentally friendly products, they can also be matted into sheets. They can be employed in both powder and fibre forms as a component of composite materials [1,2].

Cellulose, the most prevalent biopolymer and nanomaterial, may be found in great abundance in natural fibres. The importance of cellulose is growing in the modern era because of its sustainability, biocompatibility and biodegradability. Even though the natural world is full of fibrous materials, particularly those that are cellulosic like cotton, wood, grains and straw, only a very few of them can be used to make textiles or for other industrial uses. Modern man is being tempted to use natural fibres in the creation of novel composite materials by rising environmental awareness and the current economic considerations. Other than financial factors, a fibre’s suitability for commercial use is decided by characteristics like length, strength, pliability, elasticity, abrasion resistance, absorbency and different surface characteristics [1].

Natural fibres are plentiful, made from renewable resources, inexpensive, tough, have relatively high specific strength/stiffness qualities, are low abrasive, require less energy to manufacture, are CO_2_ neutral and are less dense. These characteristics have stimulated research into using natural fibres instead of synthetic fibres as reinforcement in composites, which is also necessary to control environmental conditions [3]. Additionally, the inherent qualities of natural fibres may be advantageous to the functional qualities of composites, such as their mechanical, customised fire-retardant, superior energy-absorbing and soundproofing qualities. Natural fibres would be more eco-friendly than composites reinforced with glass fibre in that aspect. Natural fibre-reinforced polymers have mechanical qualities that are comparable to glass fibre-reinforced composites, according to research in the field. Hence, the structural parts of the automotive sector, such as panels, doors, roofs and covers, as well as furnishing items like office chairs, door panels, safety helmets, concrete beams etc., are mostly produced using natural fibre reinforced plastics [4,5,6,7,8]. Natural fibre composites have been integrated into the production of the exterior and interior automotive components. The cost of materials, sustainability and low density relative to glass fibres are the main benefits of employing natural fibres in composites [3,4,5].

In many parts of the world, municipal solid waste (MSW) landfills are the primary method for disposing of waste, with Australia accounting for 70% of the global total in 2002. One strategy to address this issue is to substitute natural materials for synthetic ones, such as reinforcements in polymer composites. However, there are several other problems and drawbacks with using natural fibres as reinforcement in polymeric composites, such as poor adhesion, variability, poor temperature resistance, hydrophilic behaviour, etc. Hence, focusing on such difficulties could enhance the composite’s quality and lead to the development of new uses for such appealing materials [3,6].

In reality, plant fibres exhibit a strong hydrophilic behaviour while glass fibres are thought to be hydrophobic. Throughout their lifetime, composite materials are frequently exposed to a variety of environmental situations; yet, under humid conditions, these reinforcing fibres’ potent hydrophilic behaviour causes them to absorb a lot of moisture from damp environments. As a result, the fibres’ structural makeup is altered, and the mechanical characteristics of composites are altered. The secret for using these bio-composites in various weathering environments is understanding how these materials behave when exposed to moisture [3].

Natural fibre composite’s moisture resistance is predicted to decline as the fibre content rises. To attain maximum strength capacity and reduce its moisture susceptibility, the amount of fibre in a composite must be kept to its ideal fibre/matrix ratio. Further study is needed to introduce chemical or physical treatments that will improve the fibre/matrix interface and increase the strength and moisture durability of composites [9,10]. Alkaline treatment, also known as mercerisation, is a chemical process in which natural fibres are submerged for a predetermined amount of time and at a predetermined temperature in a solution of sodium hydroxide (NaOH). Fibres are split from one another during the alkaline process, increasing the effective surface area that the resin can wet. Alkaline treatment also alters crystallinity, unit cell structure and fibre orientation, and increases the amount of crystalline cellulose present [11]. As a result, alkaline treatment raises the natural fibre’s surface roughness and strengthens the mechanical interlock between the fibre and resin by enhancing charge transfer. Further, it is stated that the degradation resulted in a decrease in the amount of cellulose on the fibre surface at times [12]. A natural fibre composite can be stabilised against the harmful effects of UV radiation by adding a variety of chemicals [3,12].

In the present work, Zm root fibres which are grown hydroponically are taken as the material of study. Plant fibre-based investigation is done because of the urge for modern man to live in an eco-friendly environment. This is possible only by bringing green trends to Materials science. This means fabricating advantageous application-oriented composite materials which are bio-degradable and that are applicable to various industries. To begin the fabrication, the reinforcement material should be thoroughly investigated. Corn plants have a lot of applications in composite materials as many of its parts such as corn leaf fibres, corn cob powder, corn stalk fibres etc. have already been used as reinforcements. The biomasses obtained from corn such as corn starch, corn hull and corn husk are also examined for use as reinforcements in composites [13,14]. The main reason for choosing this plant fibre is the fact that corn has been used in the preparation of bioplastic. The root fibres of corn, in their unprocessed or processed form, may have a good interlocking ability with polymer matrices such as epoxy or natural rubber. In this context, the root fibres are studied using various analyses. These samples have a very low density compared to other natural fibres and thus can be employed in the preparation of light weight composites. These composites have applications in the manufacture of automotive parts, military vehicles, civil infrastructure and the sports industry where less weight is desired [15]. 

## 2. Materials and Methods

Corn, a cereal, is a vegetable and a source of flour and starch. It has industrial, pharmacological and medical uses. It can also be used to make bioplastic and biofuel. Corn cobs are used directly for fuel, to manufacture charcoal and to prepare industrial solvents. Corn stalks are processed and made into paper and wallboard. Husks are used as filler [16].

**Figure 1:**
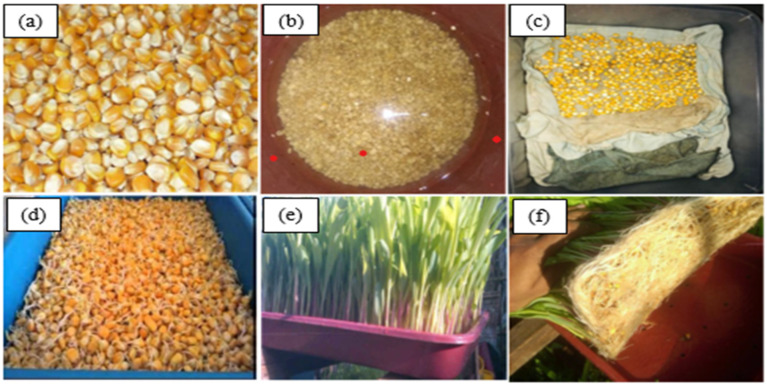
Depiction of growth of Zm root fibres: (**a**) corn seeds; (**b**) seeds soaked in water; (**c**) seeds drained and kept in a wet towel; (**d**) seeds start to germinate; (**e**) growth of corn grass; (**f**) roots of the grown grass.

The procedure for growing corn grass hydroponically is shown in Figure 1. The root fibres are harvested, dried and taken for examination after the entire growth process.

### Alkali Treatment of Fibres

**Figure 2:**
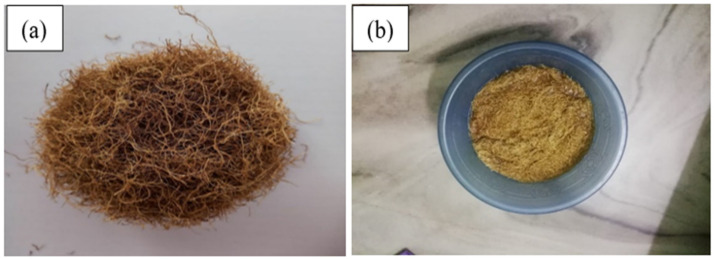
(**a**) Untreated Zm fibres; (**b**) 0.1 M alkali treated Zm fibres.

Zea mays root fibres are submerged in 0.1 M alkali solution for 10 min as shown in Figure 2b. The remaining moisture is subsequently removed from the fibres by drying them at ambient temperature.

Better mechanical bonding can be offered by alkaline treatment. This treatment improves the natural fibres’ thermostability and heat resistance. It generates the desired roughness along with increased tensile strength. Some fibres exhibit a reduction in diameter and cross-sectional area after being exposed to alkali. However, applying high concentrations and prolonged treatments leads to substantial degradation of the fibre particles themselves, which is evident from the sharp decline in tensile strength of some treated fibres [7,8].

## 3. Characterization Techniques

### 3.1. Physical Properties of Zm Root Fibres

#### 3.1.1. Diameter Using Microscope

One of the key factors in determining a natural fibre’s physical characteristics, particularly its tensile strength, is its diameter [17]. Using a compound microscope, it is possible to predict the diameter of Zm root fibres [18]. The diameters of 25 fibres from samples that are both raw and treated with alkali are measured [19,20,21]. Natural fibres typically do not have a constant diameter along their whole length due to variations in environmental conditions. As a result of its variable thickness, a natural fibre’s diameter is exceedingly difficult to quantify [14,15].

#### 3.1.2. Aspect Ratio

The strength of natural fibres is greatly influenced by their diameter and length [22]. One can calculate the aspect ratio of natural fibres by dividing their length by their diameter. It is recommended to use a natural fibre with a high aspect ratio because of its greater tensile strength [18]. Twenty fibres from Zm samples that are both raw and alkali treated are measured and tabulated for their average diameter and length. The following equation can be used to determine the aspect ratio of plant fibres:

Aspect ratio = L/D(1)

where L—average length of fibres; D—average diameter of fibres.

#### 3.1.3. Linear Density

The ratio of fibre length to mass per unit length, or vice versa, is known as the linear density. Decitex, denier, metric count etc. are used to measure linear density [23]. The linear density of fibres is employed in the Tex system, in accordance with ASTMD 1577-92, to assess their fineness. To obtain the average value, 25 Zm fibres are extracted and measured [24,25,26]. Both the untreated and alkali treated samples’ linear densities are discovered and are tabulated.

Linear density = M/L g/km(2)

where M—average mass of the fibres; L—average length of the fibres.

#### 3.1.4. Density Using Pycnometer

The density of natural fibres is frequently assessed using a pycnometer [22]. The fibre sample is simply dried at ambient temperature before use to remove the moisture content [17]. If there is still moisture in the fibre material, a vacuum desiccator can be employed to entirely remove it. The samples are then thoroughly pulverised and placed in the pycnometer to determine the density [27]. According to the ASTM D578-89 standard, using toluene as an immersion solvent, the densities of untreated and alkali treated Zm fibres are determined. Before being weighed to measure the density, the fibres must be submerged in toluene for at least two hours. The following empirical relationship governs the density of fibres.

(3) $$\rho_{\mathrm{zmf}}=\frac{(m_{2}\;-\;m_{1})\;}{(m_{3}-m_{1})-(m_{4}-m_{2})\;}=\rho_{t}$$

where, m_1_—mass of dry empty pycnometer (g); m_2_—mass of pycnometer + fibre (g); m_3_—mass of pycnometer + toluene (g); m_4_—mass of pycnometer + toluene + fibre (g); ρ_t_—density of toluene (0.867 g/cm^3^); ρ_zmf_—density of Zea mays fibre in g/cm^3^ [27,28,29,30,31,32,33].

#### 3.1.5. Thermal Conductivity using Lee’s Disc

Thermal conductivity is the capacity of a material to transport or conduct heat. Typically, the letter K is used to represent it. The term “Thermal resistivity” refers to this quantity’s reciprocal. While materials with low values of K are employed as thermal insulators, materials with high thermal conductivity are used in heat sinks. Different natural fibres have different thermal conductivities, ranging from 0.0599 W/(mK) to 0.0341 W/(mK). Using Lee’s disc experiment, it is possible to determine the heat conductivity of natural fibres. To utilise the experiment, the fibres must be formed into a disc shape. The following formula is used to get the Coefficient of thermal conductivity:

(4) $$K=\frac{m_{c}(\mathrm{dT}/\mathrm{dt})\;x}{{\mathrm{pR}}_{2}\;(T_{2}\;-\;T_{1})}\cdot \frac{R+2h}{2(R+h)}W/\mathrm{mK}$$

where K—Coefficient of thermal conductivity of the sample; m—mass of the metal disc; c—heat capacity of the metal disc; dT/dt—rate of cooling of the metallic disc; x—thickness of the sample; (T_2_ − T_1_)—temperature difference across the sample thickness; R—radius of the sample; h—thickness of the metal disc.

### 3.2. Powder X-ray Diffraction

By developing X-ray diffraction patterns of a fibre sample, XRD is a technique used to identify the crystalline and amorphous phases of the material [15,22,26]. To determine the crystallinity values of Zea mays fibres, X-ray diffraction studies of raw and alkali treated Zea mays root fibres in powder form are conducted on an X-ray diffractometer (AXS D8 Advance model, made by Bruker, Billerica, MA, USA) using Cu-K_α_ radiation with a wavelength of 1.5406 Å under ambient conditions, on a rotation limit between 3° and 135° at 2θ scale. Lynx Eye was the detector utilised in this set-up. The fibre material’s crystalline and amorphous contents are represented by the peak intensities at 22° and 16°, respectively. The following equations are used in XRD analysis to assess the crystallographic information of bio-fibres, including Percentage Crystallinity (% Cr), Crystallinity Index (CI) and Crystallite Size (CS).

(5) $$\%\;\mathrm{Crystallinity}=\frac{I_{200}}{I_{200}+I_{am}}\;\times \;100$$

(6) $$\mathrm{Crystallinity}\;\mathrm{Index}=\frac{I_{200}-I_{am}}{I_{200}}$$

where *I*_200_ and *I_am_* are the crystalline and amorphous intensities at 2θ scale close to 22° and 16° correspondingly [34,35].

(7) $$\mathrm{Crystallite}\;\mathrm{size}=\frac{K\lambda }{\beta \cos\theta }$$

where K = 0.9; λ = 1.54060 × 10^−10^ m; β = p/180 × FWHM; θ = Bragg’s angle [18].

### 3.3. FTIR Spectroscopy

FTIR analysis is carried out to determine the chemical functional groups present in the natural fibre and to verify the presence of the chemical components [36]. A Perkin Elmer Fourier Transform Infrared (FTIR) Spectrometer with a scan rate of 32 scans per minute and a resolution of 2 cm^−1^ in the wave number region of 400–4000 cm^−1^ is used to produce the FTIR spectra of the Zm fibres in the KBr matrix. The sample pieces are crushed into a fine powder using a mortar and pestle before being mixed with KBr. Then, under controlled conditions, they are pressure-pelletised to create the specimen and the FTIR spectra is recorded [37,38,39,40,41].

### 3.4. SEM—EDAX Analysis

Due to the fact that they affect the mechanical and physical qualities, morphological properties can be used to predict how natural fibres will behave before they are reinforced in composite materials [20]. Using a scanning electron microscope (SEM) with EDS, the topography and morphology of the materials’ surfaces as well as biological samples are analysed. For the purpose of elemental detection, EDS is utilised. The model employed for the sample analysis is the JEOL 6390LV, which has a range of 0.5 kV to 30 kV, a resolution of 4 nm and a 300,000× magnification [42]. The images of the surface of both the samples are captured at various magnifications.

### 3.5. TGA—DTA Analysis

In polymer composites, natural fibre reinforcement must be able to withstand heat during manufacturing and preserve its properties after being heated [36]. The analysis is performed using a Perkin Elmer Diamond TG/DTA thermal analyser. The TG sensitivity is 200 mg and DTA sensitivity is ±1000 μV. The ambient temperature is 1200 °C. The test findings will show the temperature ranges where the fibre’s various constituents, including cellulose, hemicellulose, lignin, moisture and others, begin to break down.

Kinetic activation energy (Ea) is the minimal amount of energy required to start the degradation of fibres [24]. The Coats−Redfern method is used to examine the TGA data and determines the activation energy needed for the thermal decomposition process. The first-order reaction’s mathematical relation is represented as follows using the Coats−Redfern approach:

(8) $$\log\left[\frac{-\log\left(\;1-\alpha \right)}{T^{2}}\right]=\log\frac{AR}{\beta E_{a}}[1-\frac{2RT}{E_{a}}]-\frac{E_{a}}{2.303\;RT}$$

where *T*—absolute temperature, *β*—linear heating rate, *A*—frequency factor, *Ea*—activation energy, *R*—gas constant and *α*—the fraction of decomposed sample at time t. *α* = $\frac{W_{0}-W_{t}}{W_{o}-W_{f}}$, where *W_o_*—initial sample weight (before starting the decomposition reaction), *W_t_*—sample weight at any given temperature and *W_f_*—final sample weight after completion of the reaction. A linear fitting is done to log $\left[\frac{-\log\left(\;1-\alpha \right)}{T^{2}}\right]$ versus $\frac{1000}{T}$ plot, in order to determine the activation energy [43].

### 3.6. CHNS Analysis

While CHNS analysis detects elements over the entire substrate, EDX only finds those on the surface [44]. Using a fully automated Elementar Vario EL III model CHNS/O Elemental Analyzer, the elemental composition of untreated and alkali treated Zea mays root fibres (Zm) is measured. Portions of finely powdered materials are preserved in clean containers and fed into the Analyzer with their mass weighed. The test samples are burned at high temperatures with extra Oxygen to form elemental gases, which are then measured for the quantities of carbon, hydrogen, nitrogen and sulphur using the CHNS mode [45]. Carbon dioxide (CO_2_), water (H_2_O), elemental nitrogen (N_2_) and sulphur dioxide (SO_2_) are the by-products. The CHNS/O Analyzer software aids in automatically calculating the percentage of each element contained in the sample [36,37].

### 3.7. Chemical Analysis

SITRA, Coimbatore, Tamil Nadu, India, uses particular methodologies to conduct chemical analyses on raw and alkali treated Zea mays fibres. The various components of a natural fibre are identified by their weight percentages, including cellulose, lignin, wax, ash, moisture, pectin and hemicellulose. The density is then discovered as well.

### 3.8. Tensile Strength Analysis

Natural fibres must have good tensile properties while making composites because the fibre contents that are stiffer and stronger than the polymer matrix will improve the composites’ overall tensile properties. The highest stress that a fibre can sustain before breaking is referred to as its strength [39]. The tensile qualities of untreated and alkalised Zm fibres are assessed using computer controlled tensile testing apparatus with a load cell capability of 5 kN (made by Zwick/Roell, Germany), in line with ASTM D412 standards. The samples are examined at 21 °C, 30 mm/min cross head speed and 65 ± 2% relative humidity [46]. To check the correctness of the tensile test, ten fibres (each 50 mm in length) are tested, and the computed mean values of tensile strength, elongation at break and strain rate are documented [47,48,49,50,51,52,53,54,55,56,57]. The following formula is used to determine the tensile strength of Zea mays fibres:

(9) $$T=\frac{F}{A}$$

where *F* is the force in Newtons, *A* is cross-sectional area in mm^2^ and T is the tensile strength in MPa [20,24].

In order to assess the mechanical characteristics of the fibres, including their tensile strength and percentage of elongation, stress−strain curves are obtained [56,58]. Using a microscope, the chosen fibres of Zm roots are thoroughly examined in the longitudinal direction to determine their average diameter. Further, from the SEM pictures, the thickness of the fibres is determined using the ImageJ software. Both techniques produced almost the same diameter. The cross-sectional form of Zm fibres is circular [18,29,40,51,53].

The angle created by the helical winding orientations of cellulose microfibrils is known as the micro-fibril angle (MFA). In general, the amount of cellulose and spiral angle affects a plant fibre’s strength and stiffness. Using the Global deformation equation, the micro-fibril angle (α) of Zm root fibres is assessed.

ε = ln [1 + ∆L/Lo] = −ln (cos α) (10)

where ε—strain, α—micro-fibril angle (degree) and ΔL/Lo—ratio of elongation [25,26,27,28,29,30].

Young’s modulus is the term used to describe a material’s resistance to elastic deformation under load. In other words, it demonstrates a material’s stiffness.

(11) $$E=\frac{Tensile\;Strength\;\left(\sigma \right)}{Strain\;\left(\varepsilon \right)}=\frac{F/A}{\Delta L/\mathrm{Lo}}$$

where, *F*—applied force, E—Young’s modulus of the fibre, *A*—cross-sectional area of the fibre and ΔL/Lo—ratio of elongation [58].

Elongation at break, also known as fracture strain, is the ratio of the changed length to the initial length when the test specimen is fractured. It demonstrates how fibre from plants that occurs naturally can resist form changes without cracking. Tensile testing in accordance with ISO/IEC 17,025 can be used to calculate the elongation at break [14,59].

## 4. Results and Discussions

### 4.1. Physical Properties of Zm Root Fibres

#### 4.1.1. Diameter Using Microscope

The average diameter of the fibre, which will be used as its nominal diameter, must be calculated. Random readings along the fibres are used for the measurements [32]. The size of untreated and alkali treated Zm root fibres are revealed and tallied using a microscope. The values are tabulated and are shown in Table 1.

Zm fibres may have a cross-section that is nearly cylindrical and circular [24,27]. The hollow tubular nature of the fibres lowers their density. It can therefore be used in lightweight applications and is effective as a thermal and acoustic insulator [59]. NaOH typically causes the diameter of fibres to decrease [56]. When compared to raw Zm fibres, these alkali treated Zm fibres exhibit a reduction in diameter.

The diameter of stem fibres of Cardiospermum halicababum is found to be 315.4 μm [41]. The diameter of Saccharum bengalense grass fibres is 320.47 μm [60]. These diameter values are comparable to Zm root fibres. The diameter of Jute fibres are 100–450 μm and Palmyra fibres are 20–80 μm, respectively. The smaller the diameter, the better the properties of natural fibres [36].

#### 4.1.2. Aspect Ratio

High length to diameter fibres, or those that are long and thin, provide excellent attributes, but are more expensive to create. Therefore, longer and thinner plant fibres are preferable over short and thick ones since they have greater aspect ratios. Higher aspect ratio fibres are typically stiffer. Natural fibre aspect ratios vary significantly for a number of reasons [48]. The following Table 2 compares the aspect ratios of untreated and 0.1 M alkali treated Zm root fibres.

For more effective energy transfer between the matrix and fibre filaments upon impact, a larger aspect ratio is necessary. Long, interwoven fibres assist in dispersing those forces throughout the composite structure rather than keeping them concentrated in one spot. Further, increasing fibre length often results in an increase in the load bearing capacity of the fibres; if the fibres are excessively long, they may become tangled during mixing, which would lead to poor fibre dispersion and a decrease in the effectiveness of the reinforcement as a whole.

If long fibres are used, they can be matted, spun into different forms or even compressed to achieve the necessary even format of fibres in order to have a uniform distribution of additive. The parallel arrangement of fibres prior to reinforcement might occasionally be beneficial.

#### 4.1.3. Linear Density

It has been discovered that fibres with higher linear densities will have larger fibre diameters and higher fibre moisture contents. Additionally, increasing linear density causes a decrease in the fibre’s Young’s modulus. The current work supports these observations. According to SEM and microscopic investigations, the diameter of untreated Zm fibres is higher than that of alkali-treated Zm fibres. According to a chemical examination, processed Zm fibres have lower moisture content than raw fibres. According to tensile strength analysis, treated Zm fibres have higher Young’s modulus than untreated Zm fibres [61]. Table 1 lists the linear density values for Zm fibres.

#### 4.1.4. Density Using Pycnometer

Low density is desirable in certain applications because it reduces mass and has better mechanical qualities, such as in the automotive, aviation and sporting goods industries [25,26]. Density is the primary factor considered while choosing plant fibres to replace synthetic ones. When selecting materials for various applications, weight is a significant criterion [48].

When employed in composite materials, the fibre bundles’ low density values (≤0.7 g/cm^3^) suggest great potential for lightweight construction [41,60,62]. Therefore, finding distinctive low density fibre is important [23]. Consequently, the chosen fibre’s density is evaluated both before and after treatment (Table 1). Zea mays fibres have a lower density than many other natural fibres [28]. The density value of the *Corypha taliera fruit* fibre is found to be 0.86 g/cc, which is higher than raw Zea mays fibres. The density of the raw corn husk fibres is 0.34 g/cm^3^, which is slightly less when compared to alkali treated Zea mays fibres [31].

#### 4.1.5. Thermal Conductivity Using Lee’s Disc

**Figure 3:**
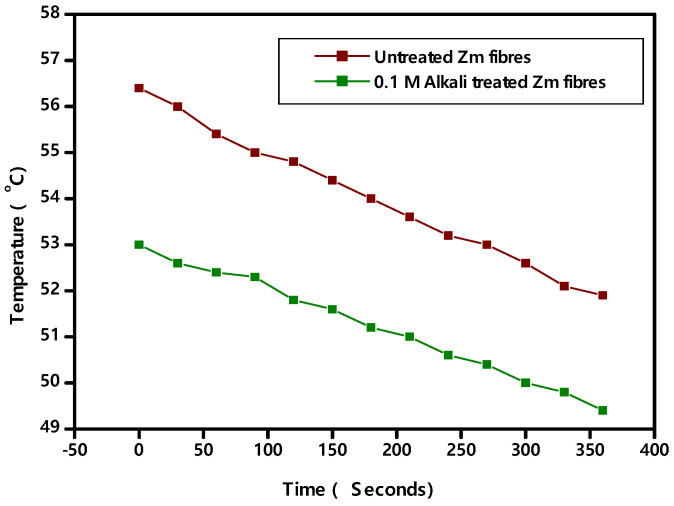
Thermal conductivity graph for Zea mays fibres.

The world’s demand for thermal insulators can be satisfied with the current supply of Zm root fibres. Using Zm fibres as thermal insulators is one way to replace non-degradable materials, or at the very least, reduce the use of non-degradable insulators. Comparing Zm fibres to other well-known thermal insulators, they exhibit minimal heat conductivity and so they can work well as one. Further research will lead to the future use of Zm root fibres, which are currently regarded as trash, as a thermal insulation material [63]. Figure 3 shows the Temperature vs. Time plot of Zm root fibre samples.

The thermal conductivity of raw Zea mays fibres is small when compared to treated fibres (Table 1). Thus, untreated Zm fibres can be used as thermal insulators with a very low conductivity value. Organic insulators, in general, are less susceptible to moisture because it accumulates in the pores rather than on the surface of the fibres. Accordingly, there is an increase in the conductivity values of the Zm root fibre samples when they are moistened, both in their raw and alkali treated forms. Indicated values were 0.0372 W/mK (raw) and 0.0793 W/mK. (0.1 M alkali treated). As a result, as the moisture level of the fibre samples increases, so does their thermal conductivity. There are certain natural fibres which have similar conductivity values. Sugarcane fibres have a thermal conductivity of 0.0469 W/mK, while Flax has 0.038–0.075 W/mK. Hemp fibres have conductivity of 0.040–0.060 W/mK and coconut fibres have 0.058 W/mK.

### 4.2. p-XRD Studies

**Figure 4:**
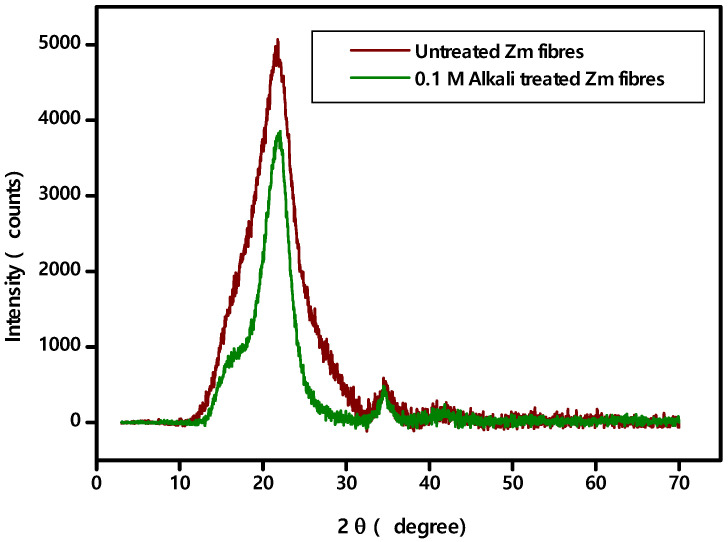
p-XRD pattern Zea mays fibres.

Calculations of % Crystallinity and Crystallinity Index showed that, when comparing alkali treated fibre to raw fibre (Table 3), crystallinity rises [35,38,57]. Because the cellulose chains become more consistently aligned, better tensile properties and greater thermal degradation temperatures are often attributed to increased Crystallinity Index values [40,64,65]. The Crystallinity Index values of Zm fibres are comparable to some natural fibres which are Napier grass strand (62.4%), Flax (70%), Sisal (75%), Coconut fibres (68%), Acacia planifrons (65.38%), Sansevieria cylindrica (60%) and Althaea Officinalis (68%) [36,54]. Figure 4 shows the X-ray diffraction pattern of both the untreated and alkali treated fibre samples.

The raw Zm fibre has a Crystallite size of 1.45 nm, and the alkali treated fibre has a Crystallite size of 2.32 nm. With chemical processing, it is reported that the Crystallite size of Zea mays fibre increases. The larger Crystallite size is advantageous since the ability of fibres to absorb water depends on the size of their crystals [47]. Additionally, when Crystallite size increases, the fibre’s chemical reactivity diminishes, increasing the mechanical strength [23,36,57]. 

Raw and alkali treated Zea mays fibres have Crystallite sizes similar to other natural fibres such as Kigelia Africana (1.73 nm), Ferula communis (1.6 nm), Azadirachta indica (2.75 nm), Coccinia grandis stem (1.91 nm), Flax fibre (2.8 nm), Corypha taliera (1.45 nm), Corn husk fibres (2.3 nm), Althaea officinalis L (2.4 nm), Cornstalk fibres (3.8 nm) and Ferula communis (1.6 nm) [24,29,30,31,54,55].

### 4.3. FTIR Spectroscopy

**Figure 5:**
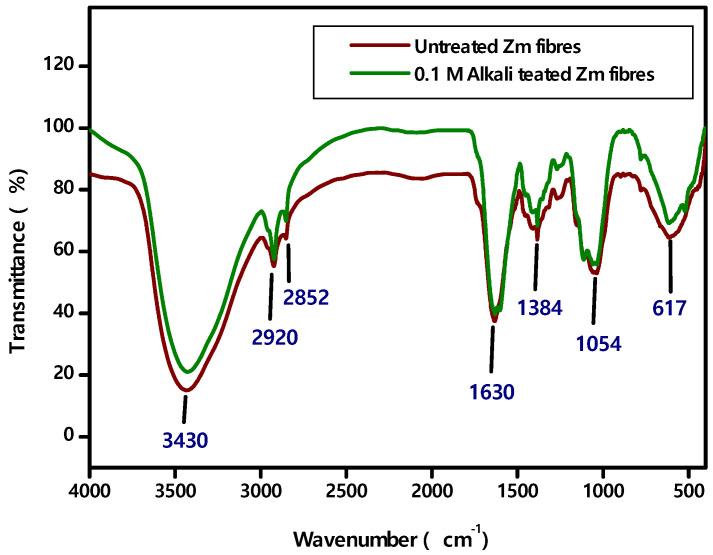
FTIR Spectra of raw and alkali treated Zm fibres.

The major fibre components (lignin, cellulose and hemicellulose) and the functional groups (ketone, alcohol and ester) may be identified using Fourier Transform Infrared (FTIR) spectroscopy. The FTIR spectra of raw and alkalised Zea mays fibres are shown in Figure 5 [40,47,58].

IR peak positions of the fibres and their characteristic Infrared absorption frequencies are given in Table 4 [38].

Again, FTIR peaks demonstrate that both Zm root fibre samples contain cellulose, hemicellulose, lignin and wax [37,38,39,40,41,63].

### 4.4. SEM—EDAX Analysis

**Figure 6:**
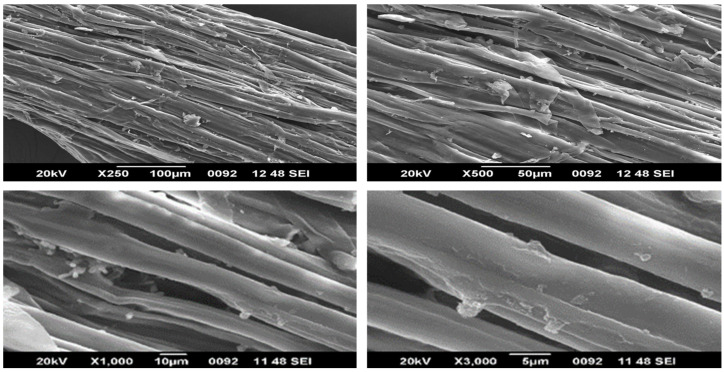
SEM images of raw Zm fibres.

**Figure 7:**
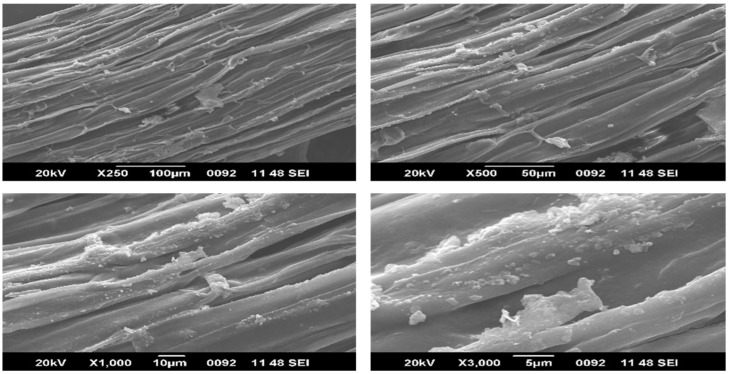
SEM images of 0.1 M alkali treated Zm fibres.

In contrast to the surface of alkali treated fibres, the surface of untreated fibres contains a significant number of gaps. On the surface of alkali treated fibres, various erratic white spots are visible from the SEM images. This attests to the fibres’ increased hemicellulose content after being treated with alkali [17]. Additionally, there is less aggregation in the untreated fibres, which would mean that the alkali treatment was successful in removing a sizable portion of the amorphous mass from the fibre surfaces, including lignin, wax, moisture and other contaminants. This investigation found that the alkali treated Zea mays fibres have a moderately increased surface roughness [18,19,27,35]. The EDX investigation has also confirmed that several encrusting compounds on the Zea mays fibre surface are carbon based [37]. Figure 6 demonstrates the natural softness and tubular shape of the raw Zm fibre with a central void (lumen) that has an approximate diameter of 414 μm. When compared to raw Zm fibres, the chemically treated fibres (Figure 7) have a diameter of around 392 μm, which is less. ImageJ software is used to determine the diameter. Composites that are stronger and stiffer typically have thinner fibres [40,51,66].

#### EDAX Analysis

**Figure 8:**
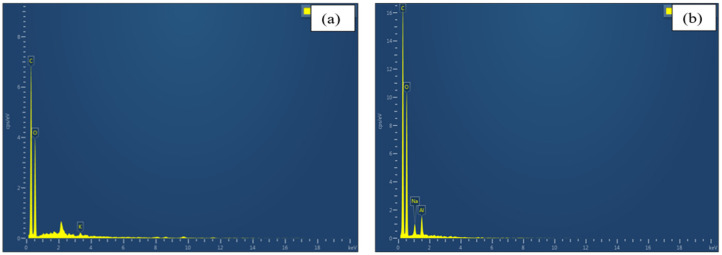
EDAX spectrum: (**a**) Untreated Zm fibres; (**b**) Alkali treated Zm fibres.

Carbon (C) and Oxygen (O) are present in higher concentrations in raw and treated Zm fibres than other elements (Table 5). A trace level of potassium was discovered in the raw fibres as the corn root fibres were being extracted from the agro-residue. In untreated fibres, significant peaks are observed for Carbon, Oxygen and Potassium as seen from Figure 8a. At minute levels, Sodium (Na) and Aluminium (Al) replace this potassium in the treated fibres (Figure 8b). Since NaOH is employed as an alkali for the chemical treatment of Zea mays root fibres, the presence of sodium supports the efficacy of the alkali treatment [23,27,47].

### 4.5. TGA—DTA Analysis

**Figure 9:**
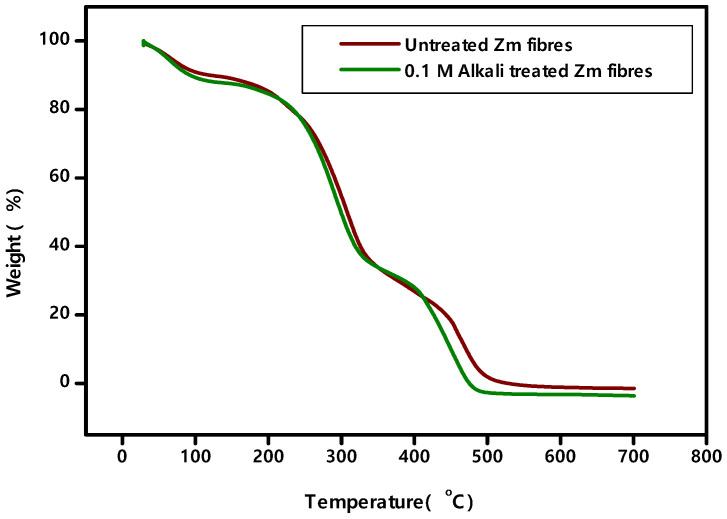
TG curves of Zm fibres.

**Figure 10:**
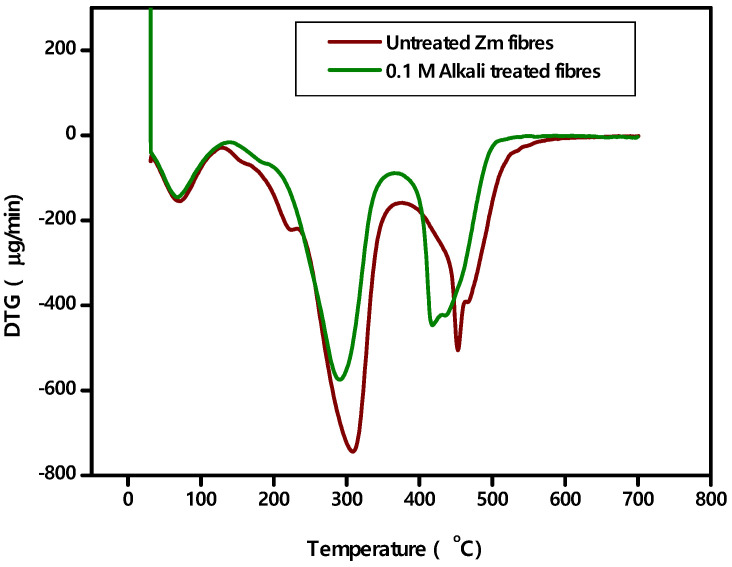
DTG curves of Zm fibres.

The mass loss of the fibres is recorded after each minute of the test, and a TG plot is generated with the temperature on the x-axis and the weight loss percentage on the y-axis (Figure 9). Additionally, using the results, the DTG curve is drawn (Figure 10).

Here is an analysis (Table 6) of the thermostability of untreated and 0.1 M alkali treated Zm fibres.

The fibre’s loss of moisture content marks the beginning of the degradation process. Hemicellulose, cellulose and some lignin decompose during the second degradation phase. The third step is when lignin begins to break down. Because lignin is a complex substance, it breaks down at high temperatures. Such details are tabulated in Table 7.

Chemical investigation has proven that untreated fibres have a higher lignin content. Thermal stability increases with increasing lignin content. As a result, untreated Zm fibres are more stable than alkali treated Zm fibres. The DTG curve is used to determine the highest deterioration peak. It is in accordance with the peak values found in the TG curve. Thus, a perfect analysis of the fibre thermal stability has been performed [22,25,52].

**Figure 11:**
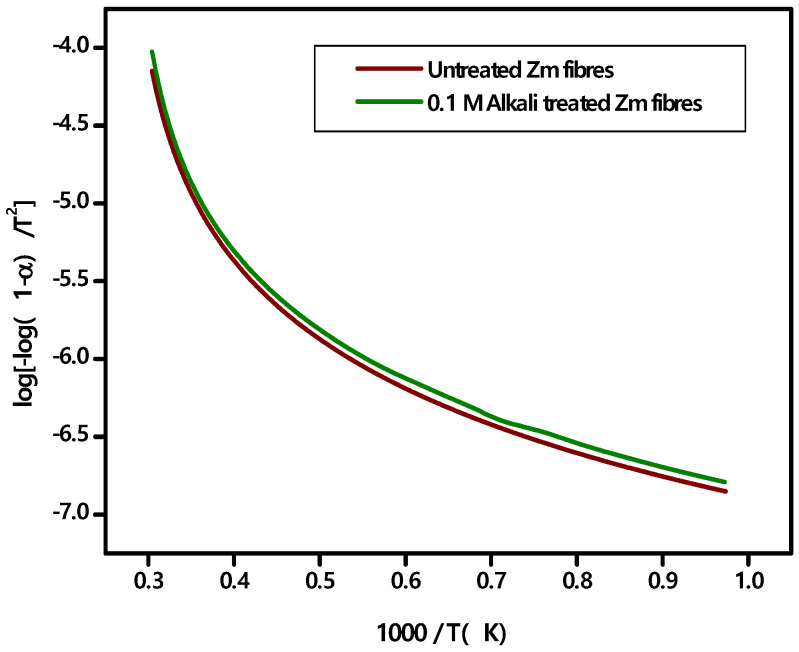
Coats-Redfern plot for raw and alkali treated Zm fibres.

Zm fibres have the thermal stability to endure the temperature of the polymerisation process for creating fibre reinforced polymer composites since the kinetic activation energy extrapolated from the Coats−Redfern plot (Figure 11) is within the acceptable range for natural materials, i.e., 60–170 kJ/mol. The elimination of the lignin and wax content increases the kinetic activation energy of alkali treated fibres [29,57].

The kinetic activation energy of Zm fibres (63.48 KJ/mol) and alkali treated Zm fibres (64.13 KJ/mol) are comparable to Indian areca fruit husk fibres (64.54 kJ/mol), Saharan aloe vera cactus (60.2 KJ/mol), Lygeum spartum (68.77 kJ/mol) and root fibres of Ficus religiosa (68.02 kJ/mol). The activation energy is higher for Derris scandens (73.2 kJ/mol), Cissusquadrangularis root fibres (74.18 kJ/mol), Prosopis juliflora bark fibres (76.72 kJ/mol) and Nendran banana fibres (89 kJ/mol) [22,25,26,32,53,55,57].

### 4.6. CHNS Analysis

According to CHNS study, raw Zm fibres have a carbon content of 41.84%, however, after being treated with alkali, the carbon content increases to 42.01% (Table 8). One of the most vital aspects to alter the mechanical and tribological qualities of the final product is high carbon content in the natural fibres. Both samples can be employed as conductive fillers in dielectric loss materials because the carbon content is around 40% in both [67].

The average compositions of carbon and hydrogen in chicken feather fibres are 47.4% and 7.2% [68]. It is found that the carbon content of coconut shell fibres and sugarcane bagasse fibres are 46.7% and 44.7%. These values are comparable to Zm fibres.

### 4.7. Chemical Analysis

While examining Table 9, it can be noticed that the alkali treated fibres have a slightly lower cellulose content. The hemicellulose content of the treated fibres marginally increases to make up for this reduction. With the treatment, it has been discovered that the amount of lignin, moisture and wax has decreased while the amount of pectin and ash has increased. Less wax and moisture content encourages the interlocking of the matrix and fibre.

High Crystallinity Index, thermal stability and tensile strength are the benefits of high cellulose content. The cellulose content of corn stover (36.40 wt.%), corn ears (38.50 wt.%), untreated corn husk (29.3 wt.%) and water soaked corn husk (30.1 wt.%) are very much less when compared to untreated (58.74 wt.%) and alkali treated (57.3 wt.%) Zea mays root fibres. The alkali treated corn husk (61.4 wt.%) and bleached corn husk (97.6 wt.%) have higher cellulose contents among the discovered corn family fibres. Other common natural fibres such as Jute (61–71.5 wt.%), hemp (70.2–74.4 wt.%), Flax (71 wt.%) and sisal (67–78 wt.%) have higher cellulose content while coir (36–43 wt.%), kenaf (31–39 wt.%) and bamboo (26–43 wt.%) have lower cellulose content [69,70,71].

### 4.8. Tensile Strength Analysis

Using the acquired stress−strain curve, the mechanical properties of the fibres, such as their tensile strength and percentage of elongation, were determined [56,58]. The fibrils are able to rearrange themselves along the direction of tensile deformation, improving the packing of cellulose chains. This is because of an increase in crystallinity, the removal of lignin and other impurities like wax and moisture, as inferred from XRD and chemical analysis of 0.1 M alkali treated Zm root fibres. Hence, alkali treated fibres have increased tensile strength as given in Table 10.

The tensile strength of untreated and alkali treated Zm fibres are comparable to Cardiospermum halicababum (20.7 ± 1.0 MPa), Saccharum bengalense grass (33 ± 1.5 MPa), raw (19.37 ± 7.72 MPa) and alkali treated aerial roots of banyan fibres (20.45 ± 12.20 MPa), Napier grass strands (13.15 MPa) and Cordia dichotoma (16.9 ± 1.3 MPa) [41,47,60]. Since the tensile strength of the Zm fibres is very low, it can be better reinforced in powdered form than in continuous form. 

There is a slight increase in the micro-fibril angle of treated fibres. These values are comparable to the micro-fibril angles of Red banana peduncle (12.64 ± 0.45°), Lygeum spartum (12.65° ± 2.85°), Thespesia populnea (13.94 ± 1.21°), Kigelia africana (13.5°) and Tridax procumbens (13.41 ± 0.64°). While it is marginally higher than aerial roots of banyan fibres (10.17 ± 1.587°), Prosopis juliflora (10.64°), Saharan Aloe vera (11.1°) and Ceiba pentandra bark fibres (9.3°). Similarly, it is hardly less than fibres of Derris scandens (17.65 ± 5.36°), Jute (17.1°), Sisal (17.9°) and Rayon (19°). Higher MFA offers enough flexibility to support the loads without tearing beyond a particular point. A plant fibre with a lower α has good mechanical properties and is a necessary component for polymer reinforcements [25,28,32,34,47,53,55].

The Young’s moduli of some common natural fibres are Cotton (5.5–12.6 Gpa), Jute (13–55 Gpa), Flax (27.6–45 Gpa), Hemp (50 Gpa) and Kenaf (53 Gpa). While comparing these values to the Zm root fibres, the Young’s modulus of Zm fibres is very lower. The elongation at break values of Zm fibres are comparable to many other natural fibres such as Date (2.73%), Elephant grass (2.5%), Flax (2.5%), Snake grass (2.87%), Ramie (2–3.8%) and Hemp (2–4%) fibres [19].

## 5. Conclusions

The novel cellulosic fibres obtained from the roots of Zea mays grown by the hydroponic method are analysed by subjecting them to various physical, chemical, structural, morphological, mechanical and thermal characterisation studies. The fibres are treated with 0.1 M of Sodium hydroxide solution. The physical properties such as diameter, density and thermal conductivity along with the characterisation studies such as p-XRD, FTIR, SEM-EDAX, TG-DTA, CHNS, Chemical analysis and Tensile strength analysis are performed on both the samples. 

The fibre material under examination is found to have a lower diameter, good aspect ratio, high thermal resistance and low density. The physical properties of alkali treated fibres are found to improve after treatment. Accordingly, the crystallinity and Crystallite size of the Zm root fibres is found to increase after treatment, as found from p-XRD analysis. FTIR analysis confirms the presence of chemical components of the natural fibres and vibrational bands associated with them. SEM images show the surface roughness of the fibres. The EDAX spectrum shows the presence of elements and their quantities on the surface of the fibre material. The presence of sodium in the surface of treated fibres validates the effect of alkali treatment on it. TGA–DTA studies show the thermal stability of the samples. Alkali treated samples have increased thermal stability. The activation energy of both the samples corresponds to many other natural fibres. From CHNS analysis, it has been established that the root fibres have high carbon content. Chemical analysis indicates that the fibres have a good cellulose content and the presence of amorphous hemicellulose content is also deduced. Due to treatment, a slight increase in hemicellulose is also seen. Specifically, the densities of both raw and treated Zm fibres are very low compared to any other natural fibres. Hence, it can be used in the preparation of lightweight composites. Both of the fibre samples have the lowest micro-fibril angle. These qualities are the essentials for a good reinforcement material. Conversely, it also has low tensile strength and Young’s modulus. Therefore it could be concluded that the material under consideration can be used in powdered form as reinforcement for epoxy as well as natural rubber composites. Alkali treatment has enhanced the characteristics of Zm root fibres to a certain extent. 

The future scope of the work is to carry out even more application-orientated analyses such as sound absorption ability and moisture absorption behaviour which can be done for the same samples. Different surface treatments can be applied rather than alkali treatment and the changes in fibre properties can be studied by repeating all of the above analyses for the new sample to find the best corn root sample to use as reinforcement in composites. The hydroponic method of growth can be replaced with any other method and the changes due to it can be analysed. Moreover, any other natural fibre can be added along with the Zm root fibres and the characteristics of this hybrid reinforcement material can be evaluated. Thus, a novel natural fibre is thoroughly investigated and it is speculated to be a good and prospective reinforcement material.

## Figures and Tables

**Table 1:** Physical properties of Zm fibres.

S.No	Samples	Diameter (µm)	Linear Density (tex)	Density Using Pycnometer (g/cm^3^)	Coefficient of Thermal Conductivity (W/mK)
1.	Raw Zea mays root fibres	345	1509	0.7694	0.02990
	Raw Zm root fibres (moistened 20%)	345	1509	0.7694	0.0372
2.	0.1 M Alkali Treated Zm root fibres	340	1384	0.5576	0.05390
	0.1 M alkali treated Zm root fibres (moistened 90%)	340	1384	0.5576	0.0793

**Table 2:** Aspect ratio of Zm fibres.

S.No	Samples	Length of the Fibre (cm)	Diameter of the Fibre (cm)	Aspect Ratio
1.	Raw Zea mays root fibres	18.5	0.0414634	446.1766
2.	0.1 M Alkali treated Zm root fibres	19.2	0.0392307	489.4126

**Table 3:** Crystallographic information from p-XRD studies.

Sample	% Crystallinity	Crystallinity Index	Crystallite Size (nm)
Raw Zm fibre	71.93	0.60	1.45
Alkali treated Zm fibre	79.47	0.74	2.32

**Table 4:** FTIR Vibrational band assignment of raw and alkali treated Zm fibres.

Wavenumber (cm)^−1^		Vibrational Band Assignments
Raw Zm Fibres	0.1 M Alkali Treated Zm Fibres	
3434.44	3428.02	Hydrogen bonded O-H stretching of cellulose
2923.63	2922.35	C-H stretching of cellulose
2852.46	2852.25	C-H symmetric stretching vibration of hemicelluloses
2053.87	-	Wax or wax likes substance
1632.10	1631.09	Carboxyl stretch of C-O, indicating the presence of acetyl group in hemicellulose
-	1603.11	C=C group of lignin
1408.97	1410.69	C-H_2_ wagging
1384.04	1382.73	Asymmetric COC stretching of lignin
1269.02	1269.29	C-O stretching vibration of acetyl group in lignin
1109.01	1112.90	C-O lignin ring
1054.05	-	C-O stretching
1036.97	1034.25	CO group of cellulose
897.17	-	Lignin components
873.40	-	β-glycosidic linkage between monosaccharide ring
778.32	779.86	CO stretching
617.00	614.00	Out of plane bending vibration involving ring structure
-	521.55	Out of plane bending of OH

**Table 5:** Weight % and Atomic % of various elements present in untreated and alkali treated Zm fibres.

Elements	Untreated Zm Fibres		0.1 M Alkali Treated Zm Fibres	
	wt %	at %	wt %	at %
C	55.99	63.03	52.06	59.59
O	43.55	36.81	45.43	39.04
K	0.46	0.16	-	-
Na	-	-	1.13	0.68
Al	-	-	1.38	0.7

**Table 6:** Thermal study of untreated and alkali treated Zm fibres.

Type of Fibre	Temperature During Mass Loss (°C)	Mass Loss (%)	Residual Char at 700 °C
Untreated Zm Fibres	28–120	10.07	0.076
	120–345	54.27	
	345–525	35.49	
0.1 M Alkali Treated Zm fibres	28–120	11.73	0.286
	120–340	53.21	
	340–474	34.59	

**Table 7:** Mass loss at T_max_ of untreated and alkali treated Zm fibres.

Types of Fibre	Total Mass Lost (%)			Max. Temperature Limit (°C)	T_(5%)_ (°C)
	First Stage	Second Stage	Third Stage		
Untreated Zm Fibres	10.07	64.34	99.83	525	65
0.1 M Alkali Treated Zm fibres	11.73	64.94	99.53	474	60

**Table 8:** CHNS analysis of untreated and alkali treated Zm fibres.

	Sample	N%	C%	S%	H%	Sample Weight (mg)
1.	Untreated Zea Mays Root Fibre	1.76	41.84	ND	6.00	11.80
2.	Alkali Treated Zea Mays Root Fibre	1.80	42.01	ND	6.30	8.20

ND—Not detected.

**Table 9:** Chemical Analysis of Zm fibres.

Chemical Composition	Untreated Zm Root Fibres %	Alkali Treated Zm Root Fibres%
Cellulose Content	58.74	57.3
Lignin Content	19.04	16.5
Wax Content	1.37	1.12
Ash Content (on dry basis)	3.47	7.0
Moisture Content	11.86	9.2
Pectin	4.69	8.2
Hemi Cellulose	29.53	30.8
Density, g/cc	0.64	0.31

**Table 10:** Tensile Analysis of Zm fibres.

Samples	Tensile Strength (N/mm^2^ or MPa)	Microfibril Angle (α)	Young’s Modulus (Mpa)	Elongation at Break (%)
Untreated Zm fibres	10.74406	12.17°	467.13	2.3
0.1 M Alkali Treated Zm fibres	13.243	12.68 °	529.72	2.5

## Data Availability

The data presented in this study are available on request from the corresponding author.
